# Evaluation of IgM, IgA, and IgG Antibody Responses Against PCV3 and PCV2 in Tissues of Aborted Fetuses from Late-Term Co-Infected Sows

**DOI:** 10.3390/pathogens14020198

**Published:** 2025-02-16

**Authors:** Jesús Hernández, Alexandra Henao-Díaz, Mónica Reséndiz-Sandoval, Joana Ramírez-Morán, Angel Cota-Valdez, Verónica Mata-Haro, Luis G. Giménez-Lirola

**Affiliations:** 1 Laboratorio de Inmunología y Laboratorio Nacional CONAHCYT de Generación de Vacunas y Servicios de Diagnóstico (LNC-GVD), Centro de Investigación en Alimentación y Desarrollo, A.C., Hermosillo 83304, SON, Mexico; mresendiz@ciad.mx (M.R.-S.); joanacrm2210@gmail.com (J.R.-M.); 2 Grupo Bachoco, Unidad de Negocios Cerdo, Calaya 38000, GTO, Mexico; yulyalex@gmail.com (A.H.-D.); angedlcota@hotmail.com (A.C.-V.); 3 Laboratorio de Microbiología e Inmunología, Centro de Investigación en Alimentación y Desarrollo, A.C., Hermosillo 83304, SON, Mexico; vmata@ciad.mx; 4 Department of Veterinary Diagnosis and Production Animal Medicine, College of Veterinary Medicine, Iowa State University, Ames, IA 50011, USA

**Keywords:** PCV2, PCV3, reproductive failure, co-infection, fetal antibodies, transplacental infection

## Abstract

Porcine circovirus type 2 (PCV2) is a ubiquitous pathogen, and co-infections with the emerging PCV3 are increasingly reported. Both PCV2 and PCV3 have been implicated in reproductive failure, yet the diagnostic criteria for PCV3 remain under development. While fetal or neonatal antibody detection is a recognized indicator of transplacental infection in multiple species, PCV2 appears to be an exception due to the possible transfer of maternal antibodies. This study evaluated IgG, IgA, and IgM antibodies in the heart, kidney, lung, and spleen of aborted fetuses from sows co-infected with PCV2 and PCV3. PCR analysis revealed that all aborted fetuses were positive for both PCV2 and PCV3, with PCV3 Ct values being generally lower than those of PCV2, although this difference was not statistically significant. Antibody profiling showed a higher prevalence of anti-PCV3 IgM and IgA compared to anti-PCV2 IgM and IgA, particularly in the heart, kidney, and lung, while IgG responses against both viruses were similar. These findings suggest that the detection of anti-PCV3 antibodies in fetal tissues may provide supportive evidence of PCV2 and PCV3 infection and the possible involvement of these viruses in reproductive failure; however, further studies are needed to establish causation definitively.

## 1. Introduction

Porcine circoviruses (PCVs) are nonenveloped viruses with a circular and single-stranded DNA genome. Four PCV species have been described: PCV1, PCV2, PCV3, and PCV4 [1]. PCV1 has been identified as a contaminant of PK15 cells and is considered nonpathogenic. PCV4 is an emerging virus first reported in 2019 in China and subsequently detected in several countries, though its pathogenicity remains unclear [2]. In contrast, PCV2 and PCV3 are pathogenic [3,4]. PCV2 is ubiquitous worldwide and causes porcine circovirus disease (PCVD), which includes PCV2-systemic (PCV2-SD) and reproductive disease (PCV2-RD) and subclinical infection (PCV2-SI), leading to increased mortality, poor growth, and significant economic losses [4]. PCV3 has been associated with respiratory, gastrointestinal, and neurological problems [3]. Both PCV2 and PCV3 cause reproductive failure, mummified fetuses, and abortions, and co-infection may exacerbate these outcomes [4,5,6]. Numerous studies have demonstrated the transplacental transmission of PCV2, typically resulting in reproductive failure (i.e., mummifications, abortions, stillbirths, and myocardial lesions in fetuses). Vertical transmission is further supported by viremia in newborn pigs, and in situ hybridization is widely accepted for confirming PCV2-RD. Similar diagnostic criteria have been proposed for PCV3 [7].

Fetal antibodies can help confirm intrauterine infections. For instance, fetal IgM has been used to detect transplacental infections in humans [8] and lambs [9]. In pigs infected with Zika virus, fetal IgM is indicative of intrauterine infection [10]. Regarding PCV2, fetal IgM and IgG can be detected at low concentrations in serum by 70 and 84 days of gestation [11]. Experimentally, low levels of anti-PCV2 antibodies have been detected in newborn piglets [12] and inoculated fetuses [13], suggesting intrauterine infection. These antibodies appear only in fetuses inoculated at later gestational stages (75 or 92 days) and not earlier (57 days) [14]. However, Saha et al. (2014) suggested that anti-PCV2 IgG antibodies can cross the epitheliochorial placenta if sows have high antibody levels, resulting in detectable, albeit low, levels of antibodies in pre-colostral serum samples [15]. They emphasized careful interpretation of fetal or stillborn antibodies. To date, there are no reports of anti-PCV3 antibodies in fetuses.

Given the epitheliochorial nature of the porcine placenta, maternal antibodies generally do not cross the placenta barrier unless it is compromised (e.g., by viral infection). Consequently, the presence of virus-specific antibodies in fetal tissues strongly indicates that these antibodies were produced by the fetal immune system in response to an in utero infection, rather than being transferred via colostrum from the sow. Thus, this study evaluated the presence of PCV2 and PCV3 in 11 aborted sows and assessed the presence of anti-PCV2 and anti-PCV3 IgM, IgA, and IgG antibodies in the heart, kidney, lung, and spleen of their aborted fetuses.

## 2. Materials and Methods

### 2.1. Samples

Samples were collected from a 650-sow farm located in northwestern Mexico, which maintained a PCV2 vaccination program using a commercial subunit vaccine based on ORF2. Piglets were vaccinated with a single dose at 21 days of age, gilts received two doses before insemination, and sows were given a single dose at 14 days post-farrowing. The farm had previous records of PCV3 detection and was free of porcine reproductive and respiratory syndrome virus (PRRSV), *Mycoplasma hyopneumoniae*, and other reproductive pathogens like parvovirus and leptospira. Serum samples were obtained from 11 sows presenting late-term abortions (Table 1). From each sow, two aborted fetuses were selected, and heart, kidney, lung, and spleen tissues were collected, yielding a total of 22 tissue samples for each organ.

### 2.2. ELISA

ELISAs were performed as previously described [1], with minor modifications. Briefly, PCV3 CAP (2 µg/mL), PCV2 CAP (2 µg/mL), and PRRSV N protein (2 µg/mL) were individually used to coat Maxisorp ELISA microwell plates (Nunc, Thermo Fisher Scientific, Waltham, MA, USA). Proteins were diluted in coating buffer (ImmunoChemistry Technologies, Davis, CA, USA), incubated overnight at room temperature, and then blocked with General Block Buffer (ImmunoChemistry Technologies, Davis, CA, USA). Serum samples were diluted 1:100 in General Sample Diluent (ImmunoChemistry Technologies) and incubated for 30 min with gentle agitation. For fetal tissues, 1 mg of tissue was homogenized in 1 mL of PBS pH 7.4, and 50 µL of the clarified supernatant was mixed with 50 µL of PBS pH 7.4 and incubated for 2 h at room temperature with gentle agitation. After five washes, 50 µL of goat anti-porcine IgG-HRP (Polyclonal; Cat. No. 6050-05; SouthernBiotech, Birmingham, AL, USA) was added and incubated for 30 min. Subsequently, 50 µL of 3,3′,5,5′-tetramethylbenzidine (Immunochemistry Technologies) substrate was added, followed by 50 µL of 1 M H_2_SO_4_ to stop the reaction. Absorbance was measured at 450 nm using a Thermo Scientific Multiskan FC Microplate Photometer (Thermo Scientific, Waltham, MA, USA). The results are expressed as optical density (O.D.) values after subtracting the absorbance of blank controls.

### 2.3. Real-Time PCR

Total DNA was extracted from serum and tissue samples using the QIAamp DNA Mini Kit (Qiagen, Hilden, Germany), following the manufacturer’s instructions. Serum samples were pooled into five groups, while samples comprising proportional amounts of heart, lung, kidney, and spleen tissues from two fetuses per sow were analyzed individually. Real-time PCR (qPCR) assays for PCV3 or PCV2 ORF2 were performed as previously described [1]. Each 25 μL reaction consisted of 150 nM primers, 10 μL of Brilliant III Ultra-Fast SYBR Green qPCR Master Mix (Agilent Technologies, Houston, TX, USA), 10.25 μL of molecular-grade water, and 4 μL of DNA template. Amplification was carried out in a StepOne Real-Time PCR system (Applied Biosystems, Houston, TX, USA) under the following conditions: initial denaturation at 95 °C for 3 min, followed by 40 cycles at 94 °C for 5 s, 60 °C for 10 s, and 72 °C for 5 s. Samples with Cq values < 35 were considered positive for PCV3 or PCV2.

### 2.4. Statistical Analysis

Data normality was assessed using the Shapiro–Wilk and Kolmogorov–Smirnov tests; as a result, a non-parametric approach was applied for further analysis. Differences in PCV2 and PCV3 IgG antibody levels and viral DNA were evaluated using the Mann–Whitney test. All analyses were conducted with a significance level of 0.05 using GraphPad PRISM software (version 10.4.0).

## 3. Results

### 3.1. PCV2 and PCV3 Detection in Sera from Aborted Sows

Real-time PCR analysis on serum samples from 11 sows with a late-term abortion revealed that 4 of the 11 sows tested positive for PCV2, while 9 of the 11 tested positive for PCV3. The Cq values for PCV2-positive samples ranged from 30.7 to 32.0, and those for PCV3-positive samples ranged from 27.5 to 33.4 (Table 2). Samples with Cq values greater than 35 were considered negative.

### 3.2. Anti-PCV2 and Anti-PCV3 Antibodies in the Sera of Aborted Sows

**Figure 1:**
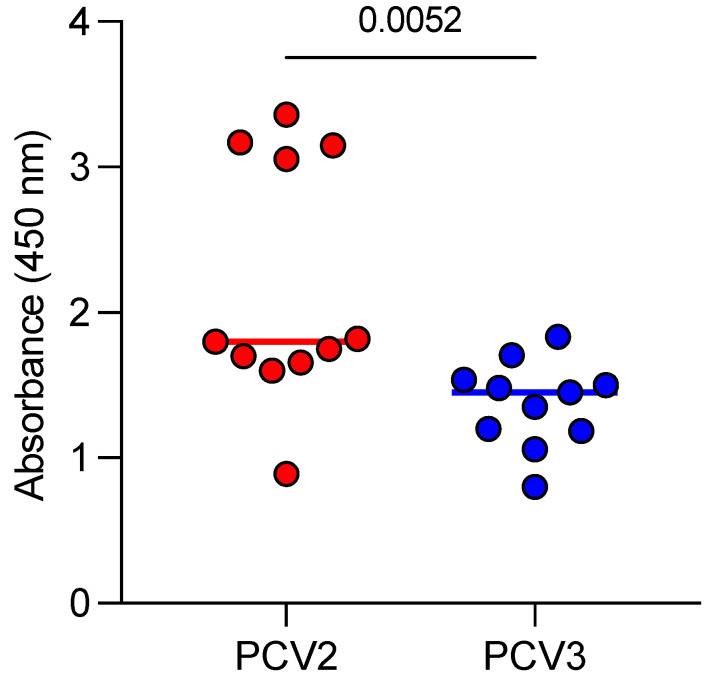
Anti-PCV2 and anti-PCV3 antibodies in the serum of sows with late-term abortions. Each data point represents an individual sow (n = 11).

All 11 sows with late-term abortions tested positive for anti-PCV2 and anti-PCV3 antibodies. For PCV2, four sows presented antibody levels greater than 3, while the remaining seven had values below 2. In contrast, all sows showed PCV3 antibody values below 2 (Figure 1).

### 3.3. Viral DNA of PCV2 and PCV3 in Aborted Fetuses

Real-time PCR was performed on a pooled sample comprising proportional amounts of heart, lung, kidney, and spleen tissues from two fetuses per sow, resulting in a total of 22 aborted fetuses being analyzed. Table 3 shows that all pooled samples were positive for both PCV2 and PCV3. The geometric mean (GM) Cq value for PCV2 was 16.62 (95% CI, 11.44–24.14), and for PCV3, it was 13.95 (95% CI, 11.53–16.87). Although the GM Cq value was numerically lower for PCV3 than for PCV2, this difference was not statistically significant (*p* > 0.05).

### 3.4. IgG, IgA, and IgM Antibodies in the Tissues of Aborted Fetuses

**Figure 2:**
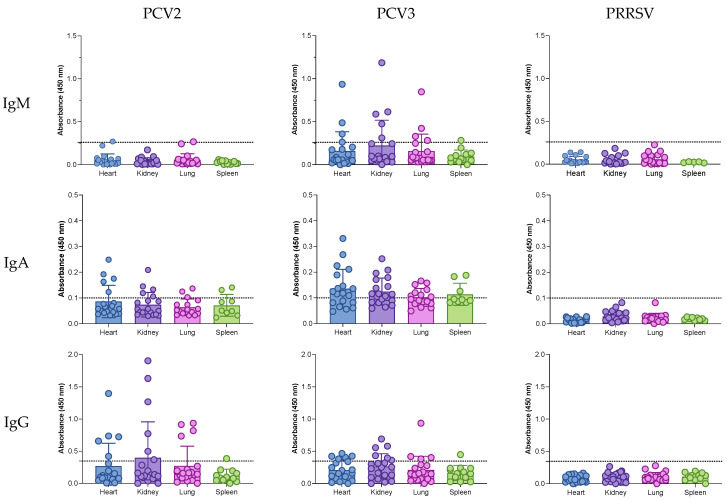
Anti-PCV2 and anti-PCV3 antibodies in the serum of aborted sows.

IgM, IgA, and IgG antibodies were evaluated in the hearts, kidneys, lungs, and spleens of the aborted fetuses (Figure 2 and Table 4). Since the farm was negative for PRRSV, the detection of anti-PRRSV antibodies served as an additional negative control for the ELISAs. The cutoff for each isotype was defined as the mean plus two standard deviations of the absorbance values.

For anti-PCV2 IgM, only a single sample from the heart and lung (1/20, 5%) tested positive, with values close to the cutoff (0.270 and 0.266, respectively). In contrast, anti-PCV3 IgM was detected in all the tissue types: 4/20 samples (20%) in the heart, 4/20 (20%) in the lung, 5/20 (25%) in the kidney, and 1/14 (7.14%) in the spleen. Anti-PCV2 IgA was detected in all tissues. Among the heart, kidney, and lung samples, 5/19 (26.3%), 7/20 (35%), and 4/17 (23.5%) were positive, respectively. In the spleen, 7/9 (77.8%) samples were positive. Anti-PCV3 IgA positivity was even higher across all tissues: 14/19 (73.7%) in the heart, 12/17 (70.5%) in the lung, 15/29 (51.7%) in the kidney, and 7/9 (77.8%) in the spleen. Anti-PCV2 and anti-PCV3 IgG responses were similar across tissues. For PCV2, positive samples were found in the heart (5/20, 25%), kidney (6/20, 30%), lung (4/20, 20%), and spleen (1/13, 7.7%). For PCV3, positivity rates were 6/20 (30%) in the heart, 6/20 (30%) in the kidney, 4/20 (20%) in the lung, and 1/13 (7.7%) in the spleen. Overall, these results indicate a predominance of IgM and IgA responses against PCV3 compared to PCV2, while IgG responses were similar for both viruses. Anti-PRRSV antibodies were not detected, confirming the specificity of the ELISA.

## 4. Discussion

In this study, farm managers noted increased reproductive failure and suspected PCV3 involvement, prompting further investigation. Under field conditions, especially in cases of late-term abortions, it is often infeasible to collect fresh fetal serum of sufficient volume or quality for analysis. Tissue sampling provides a practical alternative. This study evaluated 11 sows with reproductive failure and their aborted fetuses to determine the presence of PCV2 and PCV3 viral DNA and assess anti-PCV2 and anti-PCV3 IgM, IgA, and IgG antibodies in fetal tissues. Although PCV2 DNA was detected in the serum of only 4 of the 11 sows, PCV3 DNA was found in 9 of the 11 sows. The Cq values observed in the sow’s sera were relatively high and, as reported elsewhere [4], do not strongly implicate these viruses as the sole cause of clinical signs. In contrast, the aborted fetuses showed low Cq values for both PCV2 and PCV3, suggesting high viral loads [16]. Although PCV3 tended to show lower Cq values than PCV2, the difference was not statistically significant (*p* > 0.05). Previous reports have associated reproductive failure with PCV3 alone or co-infection with PCV2 [17,18,19,20], and our data support these observations but cannot conclusively determine which virus was primarily responsible for the reproductive failure. It appears that both viruses may have contributed, as the sows experienced abortions despite varying parities and PCV2 vaccination histories. Given that PCV2 vaccination is generally effective at controlling PCV2-associated reproductive failure, the substantial presence of PCV3 suggests that PCV3 infection may affect vaccine efficacy or contribute independently to reproductive problems. This aligns with our previous data showing PCV3 interference with PCV2 antibody responses in oral fluids [21], although other authors have reported no such effects [22].

Previous studies have also demonstrated that tissue-based assays can reliably detect immunological responses, including local antibody production, which are indicative of in utero infection [10,11]. However, the interpretation is complicated for PCV2 because maternal IgG can cross the placenta under certain conditions [15]. In this study, we assessed anti-PCV2 and anti-PCV3 IgM, IgA, and IgG in fetal heart, kidney, lung, and spleen tissues. All three isotypes were detected in at least one tissue, with a higher frequency of anti-PCV3 IgM and IgA compared to PCV2. Several potential explanations arise from these findings:

(i) The data may indicate a true intrauterine co-infection with PCV2 and PCV3.
(ii) The higher frequency of anti-PCV3 IgM compared to anti-PCV2 IgM suggests active PCV3 infection within the fetus.
(iii) The higher frequency of anti-PCV3 IgA further supports ongoing PCV3 infection.
(iv) An initial PCV2 infection, controlled by the vaccination program, may have reduced the virus load and, consequently, the induction of IgM. Some IgG antibodies could represent maternal transfer, especially if PCV2 infection altered the placenta.

These scenarios collectively imply that PCV2 vaccination, while effective at controlling clinical manifestations, may not fully prevent co-infections or their consequences. PCV2 could have predisposed the fetuses to or facilitated PCV3 infection, contributing to reproductive failure. It is also important to mention that reproductive events can occur in farms that are negative for PRRSV, influenza virus, or *Mycoplasma hyopneumoniae* with a PCV2, parvovirus, *Leptospira* spp., and *Erysipelothrix rhusiopathiae* vaccination program, even without apparent environmental causes. The results of this study suggest that the detection of antibodies against PCV3 and PCV2 in aborted mummies could explain the reproductive episodes on the farms. This strategy could offer a differential diagnosis to explain reproductive failure not associated with other common pathogens.

Previous studies have confirmed co-infections of PCV2 and PCV3 in reproductive disorders [6,17,19,20]. Many of these studies incorporate qPCR and in situ hybridization data, along with histopathological findings, to implicate PCV3 in reproductive failure. Some authors have recommended applying diagnostic criteria for PCV3 similar to those used for PCV2 [7]. Our results support considering fetal antibodies, particularly anti-PCV3 IgM and IgA, as indicators of in utero viral infection and as having possible involvement in reproductive failure, especially in cases where other potential reproductive pathogens are ruled out.

It is also important to remark that we primarily focused on detecting the presence of PCV2 and PCV3 using qPCR, rather than characterizing the full genome or subgenotypes of PCV2. Further genotyping to determine whether circulating viruses belong to PCV2a, 2b, 2d, or other subtypes would offer additional insight into the degree of vaccine–virus matching and potential immune escape.

This study was conducted under field conditions, which introduced several constraints that should be considered when interpreting the results. First, obtaining high-quality fetal samples for comprehensive pathological or immunohistochemical evaluations was difficult because the aborted fetuses were often in suboptimal conditions at the time of collection. Second, not all fetuses within each litter were non-viable; however, the overall event was categorized as an abortion scenario by farm management due to the premature expulsion of fetuses. Third, it was not possible to determine the precise timing or sequence of PCV2 and PCV3 infections, highlighting the need for future studies to elucidate whether a certain virus plays a more critical role in reproductive failure during co-infections. Fourth, this study relied on Cq values, which provide only a relative estimate of viral load. Finally, only two fetuses per sow were analyzed, limiting the ability to correlate maternal viral loads or antibody levels with fetal infection status at an individual level. Despite these limitations, our findings contribute to a deeper understanding of the complex interplay between PCV2 and PCV3 in porcine reproductive disorders and highlight the importance of further investigations to elucidate their pathogenic mechanism and optimize control strategies.

Overall, despite multiple attempts, establishing an experimental reproductive model for PCV3 infection and PCV2/PCV3 co-infection remains challenging. As a result, field-based studies are important for investigating these viruses under real-world conditions. In this case, the farm experienced reproductive problems even though other common reproductive pathogens, including PRRSV, PPV, and Leptospira, were ruled out. While definitive confirmation of co-infection ideally requires a sow infection model under controlled experimental conditions, our findings, consistent with other field studies [17,18,19,20], strongly suggest that PCV2 and PCV3 play significant roles in reproductive failure.

## 5. Conclusions

We acknowledge that the in situ detection of viral antigens in fetal tissues could provide valuable spatial information on the localization of PCV2 and PCV3 and offer more unequivocal evidence of their involvement in reproductive failure. However, under our field conditions, fetal tissue quality, particularly from mummified fetuses, was inadequate for reliable in situ analyses. Instead, we employed qPCR and serological assays to detect viral DNA and specific antibodies (IgG, IgA, and IgM) in multiple fetal tissues. Notably, given the epitheliochorial nature of the porcine placenta, the detection of virus-specific antibodies in fetal tissues strongly indicates that these antibodies were produced in fetuses in response to an in utero infection. Although these methods support PCV2/PCV3 infection and their potential role in reproductive failure, they are not definitive. Future studies with optimally preserved tissues could further clarify these findings.

## Figures and Tables

**Table 1:** Clinical characteristics of aborted sows.

	Age (Years)	No. of Parities	No. of Aborted Pigs	No. of Pigs Born Alive	No. of Vaccinations Against PCV2
Sow 1	1.4	2	15	3	3
Sow 2	2.5	5	2	15	6
Sow 3	1.8	3	2	16	4
Sow 4	2.5	5	3	17	6
Sow 5	1.4	2	3	14	3
Sow 6	1.4	2	5	5	3
Sow 7	1.8	3	2	16	4
Sow 8	1.4	2	2	18	3
Sow 9	2.5	5	1	12	6
Sow 10	1.0	1	2	8	2
Sow 11	2.5	5	3	15	6

**Table 2:** Viral DNA of PCV2 and PCV3 in the serum of sows with late-term abortions.

	Sow 1	Sow 2	Sow 3	Sow 4	Sow 5	Sow 6	Sow 7	Sow 8	Sow 9	Sow 10	Sow 11
PCV2	30.7	Neg	32.0	32.0	32.0	Neg	Neg	Neg	Neg	Neg	Neg
PCV3	Neg	Neg	32.0	27.5	28.8	31.5	32.0	33.4	31.29	29.55	33.25

“Neg: negative” indicates a negative result. Cq values > 35 are considered negative.

**Table 3:** Viral DNA of PCV2 and PCV3 in aborted fetuses.

	F1	F2	F3	F4	F5	F6	F7	F8	F9	F10	F11
PCV2	9.56	8.86	14.54	7.81	9.54	26.81	27.5	15.26	30.79	29.23	28.75
PCV3	12.89	11.43	27.45	12.15	12.6	12.56	9.74	16.43	16.29	11.4	16.83

Cq values are provided for PCV2 and PCV3; values >35 are considered negative. Each column (F1–F11) represents a pooled sample containing proportional amounts of heart, lung, kidney, and lung from two fetuses originating from a single aborted sow.

**Table 4:** Percentages of samples with IgM, IgA, and IgG anti-PCV2 and PCV3 antibodies in tissues of aborted fetuses.

	Heart	Kidney	Lung	Spleen
		IgM		
PCV2	5 (1 of 20)	0	10 (2 of 20)	0
PCV3	20 (4 of 20)	35 (7 of 20)	25 (5 of 20)	7.1 (1 of 14)
IgA				
PCV2	26.3 (5 of 19)	25 (5 of 20)	17.4 (3 of 17)	22.2 (2 of 9)
PCV3	65 (13 of 20)	65 (13 of 20)	47 (8 of 17)	33.3 (3 of 9)
IgG				
PCV2	25 (5 of 20)	30 (6 of 20)	20 (4 of 20)	7.7 (1 of 13)
PCV3	30 (6 of 20)	30 (6 of 20)	20 (4 of 20)	7.7 (1 of 13)

## Data Availability

Data are contained within the article.
